# 治疗效果显著的1例AFP增高的原发性肺鳞癌病例报道

**DOI:** 10.3779/j.issn.1009-3419.2021.101.50

**Published:** 2022-01-20

**Authors:** 青 陈, 衍博 王, 文杰 张, 晨 王, 俊程 尹, 其森 郭

**Affiliations:** 1 250117 济南，山东第一医科大学附属山东省肿瘤医院肿瘤内科，山东第一医科大学 Department of Oncology, Shandong Cancer Hospital and Institute, Shandong First Medical University and Shandong Academy of Medical Sciences, Jinan 250117, China; 2 255400 淄博，淄博市齐都医院 Qidu Hospital of Zibo City, Zibo 255400, China; 3 261000 潍坊，潍坊医学院 Weifang Medical University, Weifang 261000, China

**Keywords:** 甲胎蛋白, 原发性肺鳞癌, 安罗替尼, 免疫治疗, 疗效评价, Alpha-fetoprotein, Primary lung squamous carcinoma, Anlotinib, Immunotherapy, Efficacy evaluation

## Abstract

**背景与目的:**

产生甲胎蛋白（alpha-fetoprotein, AFP）的原发性肺鳞癌很少见，迄今为止只有4例相关报道。AFP增高的具体原因和治疗产生AFP原发性肺鳞癌的有效方案目前还不清楚。现报告1例产生AFP的肺鳞癌诊断和治疗经过，为后续临床工作提供参考价值。

**方法:**

回顾性分析山东省肿瘤医院2020年10月23日收治的1例AFP增高的原发性肺鳞癌患者的诊断、治疗过程并进行文献复习。

**结果:**

患者男性，52岁，初始诊断：右肺上叶鳞癌T4N3M0、IIIc期纵隔淋巴结转移肺内多发转移。肿瘤标记物以血清异常增高的AFP为主。两线化疗快速进展后，三线予以安罗替尼+卡瑞丽珠单抗方案治疗，2个周期和4个周期后疗效评估分别为部分反应（partial response, PR）和病情稳定（stable disease, SD）。第5个周期治疗后因消化道出血更换为白蛋白紫杉醇+卡瑞丽珠单抗方案治疗，病情得到持续控制。

**结论:**

案例报道中产生AFP的原发性肺鳞癌患者对安罗替尼联合免疫治疗反应良好，可为今后临床工作和研究AFP增高的原发性肺鳞癌带来指导意义。

## Abstract (en)

**Background and objective:**

Primary lung squamous carcinoma that produces alpha-fetoprotein (AFP) is rare and only four related cases have been reported so far. The specific reasons for elevated serum level of AFP and effective treatment regimens for AFP-producing lung squamous carcinoma are not clear. This paper reports the diagnosis and treatment of AFP-producing lung squamous carcinoma so as to provide some references for similar cases in clinical practice.

**Methods:**

The diagnosis and treatment of an AFP-producing lung squamous carcinoma patient admitted to the Shandong Cancer Hospital on October 23, 2020 was retrospectively analyzed, and literatures were reviewed.

**Results:**

A 52-year-old male patient was diagnosed as T4N3M0 stage, IIIc right upper lobe lung squamous cell carcinoma with mediastinal lymph node metastasis and multiple metastases in the lung. The main tumor marker was abnormally increased serum AFP. After the rapid progression of two lines chemotherapy, the patient was given anlotinib combined with carrizumab as third-line treatment. The efficacy evaluation reached to partial response (PR) and stable disease (SD) after 2 and 4 cycles of treatment, respectively. The treatment regimen was replaced with albumin paclitaxel plus carrizumab due to gastrointestinal bleeding after the fifth cycle. The patient's condition was under continuous control.

**Conclusion:**

The AFP-producing lung squamous carcinoma patient had a good response to anlotinib and immunotherapy in the case report, which may provide some guidances for the clinical practice and the research on AFP-producing lung squamous carcinoma.

## 1 临床资料

52岁男性，因“咳痰带血、呼吸困难伴胸背部疼痛不适2月”，于2020年8月8日就诊于泰安市中心医院。2个月内体重下降5 kg，吸烟指数300年支，既往体健。听诊右肺呼吸音粗，左肺呼吸音清。血清甲胎蛋白（Alpha-fetoprotein, AFP）和神经元特异性烯醇化酶（neuron-specific enolase, NSE）的血清水平分别为1, 093 ng/mL和20.54 ng/mL（图 1A）。胸部计算机断层扫描（computed tomography, CT）提示：右肺门恶性占位并纵隔淋巴结转移可能；右肺多发小结节，转移？（图 2A）。2020年8月20日行支气管检查，病理：（右下叶内基底段支气管）小细胞肺癌，结合免疫组化，倾向于低分化鳞状细胞癌可能性大，形态学不除外高级别神经内分泌癌。免疫组化：CK（-），Syn（-），CgA（-），CD56（-），TTF-1（-），Ki-67（阳性指数约95%），CK8/18（-），EMA（部分核旁点状+），Vimentin（-），CD20（-），CD3（-），Myogentin（-），MyoD1（-），TdT（-），S-100（-），HMB45（-），CK5/6（-），P63（-），P40（-），CD117（-），SALL-4（-），GPC3（-）。2020年9月4日会诊病理（齐鲁医学检验所，会诊号：H20204771）：标本挤压，形态不清晰，免疫组化表达不典型，倾向低分化鳞状细胞癌，请结合临床，排除小细胞癌。必要时重新取检。患者接受吉西他滨+顺铂方案化疗2个周期（吉西他滨1.6 g，d1，d8；顺铂30 mg，d1-d4；*q3w*）。化疗2周期后查血清AFP水平升高至1, 876.10 IU/mL（图 1B），复查胸部CT示：右肺上叶癌、纵隔淋巴结肿大治疗后所见；右侧胸腔积液。疗效评估：病情进展（progressive disease, PD）（图 2B）。患者于2020年10月23日入我院查血清AFP和NSE水平分别为2, 245 ng/mL和20.38 ng/mL（图 1C）。包括β-绒毛膜促性腺激素（β-human chorionic gonadotrophin, β-HCG）在内的其余肿瘤标记物均在正常范围内。乙型肝炎检测全阴性。2020年10月28日行正电子发射计算机断层显像（positron emission tomography-computed tomography, PET/CT）：右肺上叶周围型肺癌并右肺门、纵隔多发淋巴结转移并右肺门结构受累；右肺多发结节，考虑转移；肝脏、睾丸等脏器未见高代谢。头颅磁共振正常。会诊泰安市中心医院病理（会诊号：H2020-6658）：（右肺下叶内基底段支气管活检）恶性肿瘤，不排除低分化鳞状细胞癌，但形态不典型。再次行支气管镜检查及活检，镜下可见右中间支气管黏膜肿胀隆起，表面糜烂覆坏死物（图 3）。病理：（右肺纤支镜活检）组织挤压，结合病史及免疫组化，倾向低分化鳞状细胞癌，免疫组化：CKpan（part+）、P40（+）、Syn（-）、CgA（-）、CD56（-）、TTF-1（-）、CK7（-）、LCA（-）、Ki-67（+80%）（图 4）。患者因经济原因拒绝行基因检测及程序性死亡受体配体1（programmed cell death ligand-1, PD-L1）检测。结合临床表现、肿瘤标记物和多次病理结果，不排除合并神经内分泌癌成分，给予二线依托泊苷+卡铂方案化疗2个周期（依托铂苷0.1 g，d1-d3；卡铂300 mg，d2；*q3w*）。1周期化疗后第2天，患者突然出现意识模糊、神志淡漠。血常规检查白细胞（23.58×109/L）和中性粒细胞（19.79×10^9^/L）升高。临床实验室临界值显示钠（163 mmol/L）、钙（4.86 mmol/L）、磷（1.63 mmol/L）和氯（113 mmol/L）升高。肾功能检查显示肌酐（161 μmol/L）、尿酸（1, 046 μmol/L）和尿素氮（16.6 mmol/L）升高。结合临床表现及实验室指标，考虑为溶瘤综合征，予以连续血液净化、留置导尿和营养支持治疗后患者全身症状好转，肾功能指标恢复正常。2个周期后复查血清AFP水平为1, 701 ng/mL（图 1D）。复查胸部CT：右肺癌并右肺门及纵隔淋巴结转移，较前（2020年11月24日）增大；右肺多发结节，考虑转移，较前增大。疗效评价：PD（图 2C）。给予患者三线安罗替尼+卡瑞丽珠单抗治疗（安罗替尼10 mg，d1-d14；卡瑞丽珠单抗200 mg，d1；*q3w*）。2个周期后复查血清AFP水平下降至983.4 ng/L（图 1E）；复查CT提示右肺癌并右肺门及纵隔淋巴结转移治疗后改变，较前（2020年12月17日）好转；右肺多发结节，考虑转移，较前略缩小。疗效评价：部分缓解（partical response, PR）（图 2D）。4个周期后血清AFP水平降至正常（图 1F）。复查胸部CT：右肺癌并右肺门及纵隔淋巴结转移治疗后，较前（2020年11月19日）好转；右肺多发结节，考虑转移，较前部分变化不显著，部分饱满，建议观察。患者原发病灶基本稳定，纵隔淋巴结较前缩小，评估病情较前好转，综合评估疗效：疾病稳定（stable disease, SD）（图 2E）。在第5个周期治疗后，患者因消化道出血停用安罗替尼，调整为白蛋白紫杉醇+卡瑞丽珠单抗方案治疗（白蛋白紫杉醇400 mg，d1；卡瑞丽珠单抗200 mg，d1；*q3w*）。患者病情得到控制，影像学结果和血清AFP水平持续稳定。目前患者一般情况良好，继续治疗中。

**图 1:**
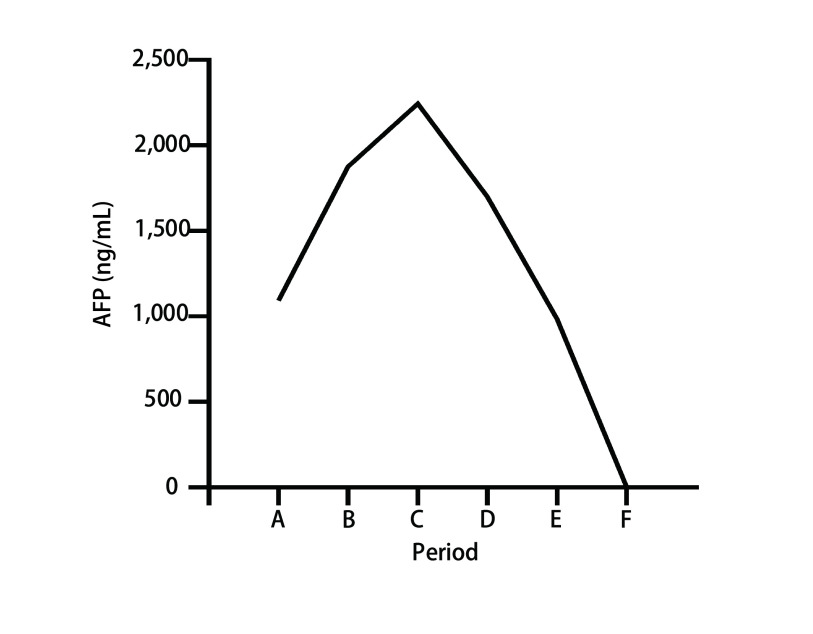
血清AFP水平。A：治疗前；B：2个周期吉西他滨+顺铂治疗后；C：入院时；D：2个周期依托泊苷+卡铂化疗后；E：2个周期安罗替尼+卡瑞丽珠单抗治疗后；F：4个周期安罗替尼+卡瑞丽珠单抗治疗后。 The serum level of AFP. A: Prior treatment; B: After 2 cycles of gemcitabine and cisplatin treatment; C: On admission; D: After 2 cycles of etoposide+carboplatin; E: After 2 cycles of anlotinib+carrizumab; F: After 4 cycles of anlotinib+carrizumab.

**图 2:**
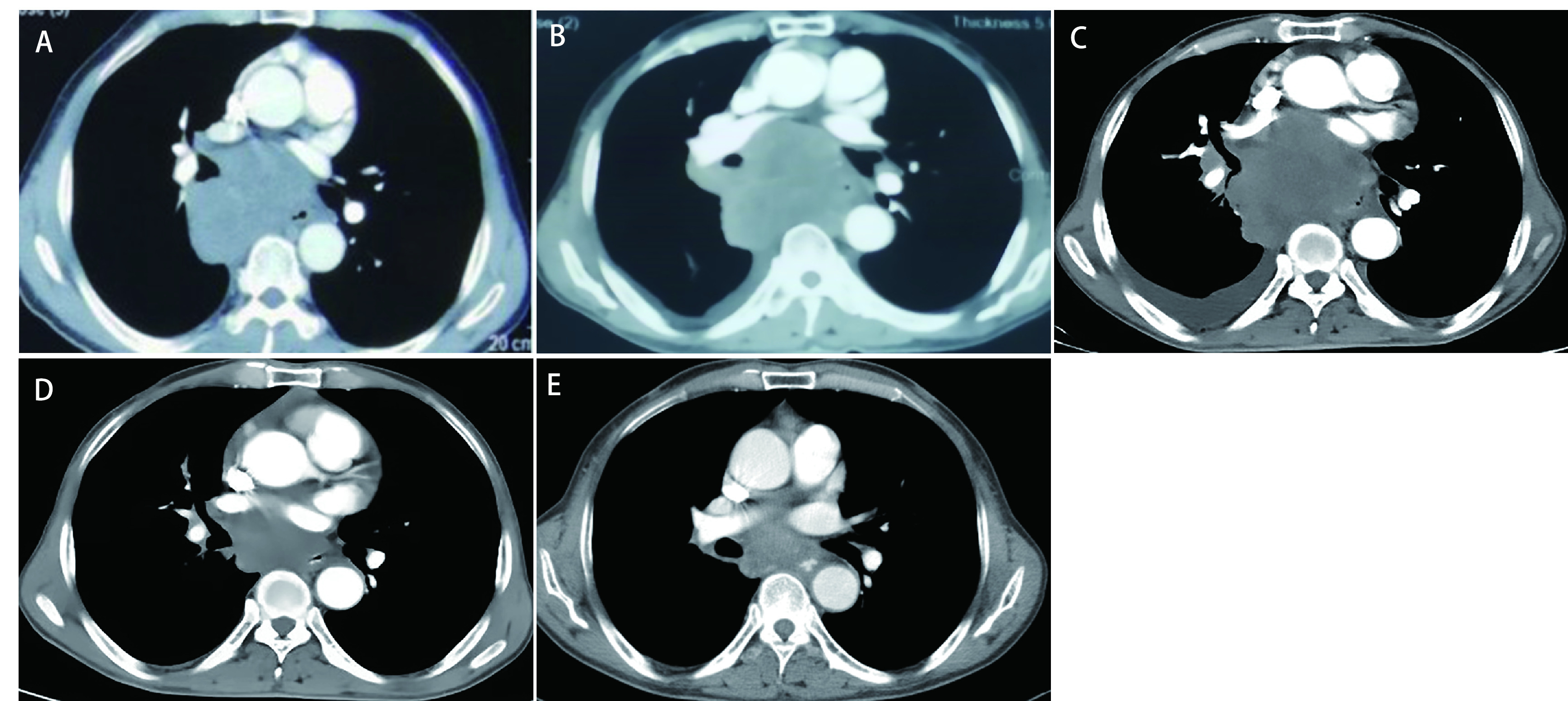
不同治疗时期的图像。A：治疗前；B：2个周期吉西他滨+顺铂化疗后；C: 2个周期依托泊苷+卡铂化疗后；D：2个周期安罗替尼+卡瑞丽珠单抗治疗后；E：4个周期安罗替尼+卡瑞丽珠单抗治疗后。 Images of different treatment periods. A: Prior treatment; B: After 2 cycles of gemcitabine+cisplatin; C: After 2 cycles of etoposide+carboplatin; D: After 2 cycles of anlotinib+carrizumab; E: After 4 cycles of anlotinib+carrizumab.

**图 3:**
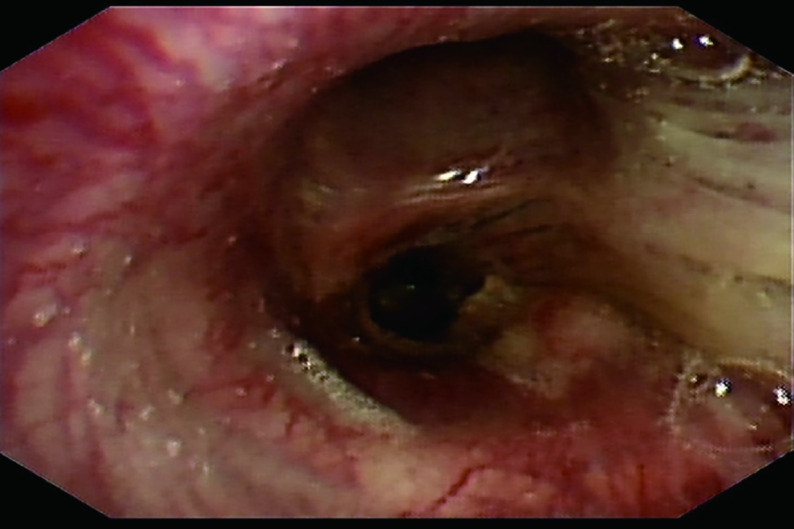
支气管镜下图像。右中间支气管粘膜肿胀隆起，表面糜烂覆坏死物。 An image under bronchoscope. The mucosa of the right middle bronchus was swollen and the surface was eroded and covered with necrosis.

**图 4:**
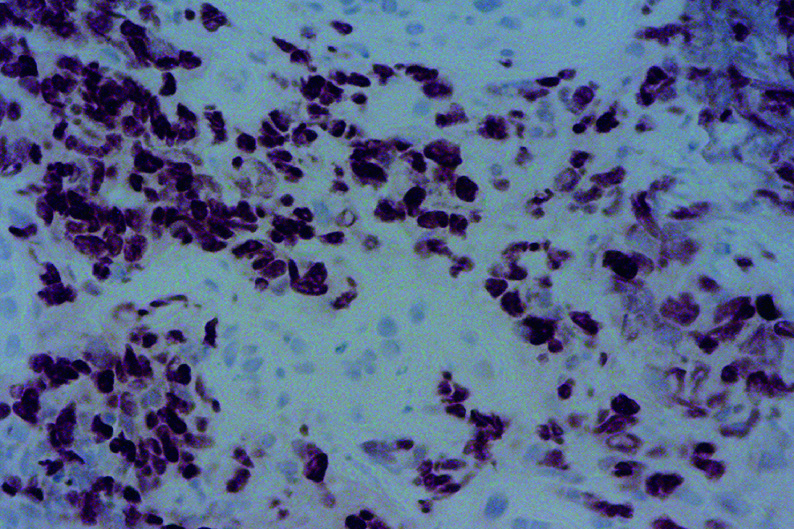
免疫组化：P40阳性（×20） Immunohistochemistry staining: P40 was positive (×20)

## 2 讨论

AFP是一种在人胚胎、肝脏、卵黄囊和胃肠道细胞中合成的血清蛋白^[1]^。AFP是诊断和预后肝癌和睾丸癌的可靠标志物，也可见于胃癌和胰腺癌^[2-5]^。Corlin等^[6]^首先报道了AFP异常升高的支气管癌，随后陆续有产生AFP的原发性肺癌的报道，但其中大多数病理类型为肺腺癌。据我们所知，到目前为止只有4篇关于产生AFP肺鳞癌的报告^[7-10]^。这4例患者的年龄从16岁-64岁，其中3例患者的总生存时间不超过2年。同样，我们的患者52岁，在很短的时间内病情迅速进展。不难发现产生AFP的原发性肺鳞癌具有恶性程度高、疾病进展快、生存期短的特点，如果治疗不当，患者会在短时间内死亡。

原发性肺癌AFP异常升高的真正机制尚不清楚。Okunaka等^[11]^回顾了24例产生AFP的肺癌，系统分析了原发性肺癌AFP升高的可能原因。首先，肺脏与肝脏一样均起源于前肠内胚层，因此可能也像肝脏一样保留了产生AFP的能力；其次，肝细胞癌异位到肺脏可导致AFP增加；此外，性腺外生殖细胞癌综合征也可能导致AFP水平升高。在今后的临床研究中，探索原发性肺癌AFP升高的真正原因，从而找到产生AFP的肺癌的最佳治疗方法是需要解决的课题。

在我们的病例中，患者的肿瘤标志物主要以血清AFP水平异常增高为主，随着治疗的进展及患者病情变化，血清AFP水平上下波动。这表明AFP可能是检测AFP异常增高的原发性肺癌的可靠标志物。

病例报道中患者在接受二线化疗后病情并未得到控制，表明AFP异常增高的原发性肺鳞癌可能对化疗反应极差。三线安罗替尼联合卡瑞丽珠单抗治疗2个周期后评估疗效为PR，4个周期后评估疗效为SD，血清AFP水平逐渐恢复正常，病情得到长期控制。可见，安罗替尼联合免疫检查点抑制剂在该患者中疗效显著。安罗替尼作为一种新型的、口服的多靶点抗血管生成药物，在小细胞肺癌和非小细胞肺癌三线治疗均获批适应证^[12, 13]^。此外，Basse等^[14]^报道了1例产生AFP的原发性肺癌，在使用免疫检查点抑制剂治疗后达到了PR，即使PD-L1表达阴性。Pasricha等^[15]^对6例AFP增高肺癌患者进行了PD-L1的免疫组化检测。其中4例PD-L1表达 > 1%，2例PD-L1表达 > 50%。这提示我们无论PD-L1表达如何，免疫治疗可能对产生AFP的原发性肺癌的治疗均有效果，PD-L1表达在AFP增高的原发性肺癌中很常见。安罗替尼与免疫检查点抑制剂在抗肿瘤过程中发挥协同作用。一方面，异常的肿瘤新生血管可以阻断周围T细胞向肿瘤组织的积累和浸润。安罗替尼可以使肿瘤血管正常化，改善肿瘤的免疫微环境；另一方面，内皮细胞表达的PD-L1的减少可引起血管内皮细胞生长因子受体-2（vascular endothelial growth factor receptor 2, VEGFR-2）的增加，因此PD-L1对肿瘤血管生成具有潜在的调节作用^[16-18]^。有报道^[19]^显示，安罗替尼联合免疫检查点抑制剂可使二线治疗失败的晚期非小细胞肺癌患者的客观缓解率（objective response rate, ORR）为18.8%，疾病控制率（disease control rate, DCR）为79.2%，无进展生存时间（progression free survival, PFS）为6.7个月（95%CI: 6.13-7.24）。因此，安罗替尼联合免疫检查点抑制剂治疗可能是AFP增高的原发性肺鳞癌有效的治疗手段。

总之，以恶性程度高、进展快为特征的产生AFP的原发性肺鳞癌在临床上很少见。安罗替尼联合免疫治疗可能是产生AFP的原发性肺鳞癌的合适方案。需要进一步的研究来解释AFP升高的机制，并为产生AFP肺癌建立有效的治疗方法。

## References

[1] Gitlin D, Perricelli A, Gitlin GM (1972). Synthesis of α-fetoprotein by liver, yolk sac, and gastrointestinal tract of the human conceptus. Cancer Res.

[2] Patel P, Balise R, Srinivas S (2012). Variations in normal serum alpha-fetoprotein (AFP) levels in patients with esticular cancer on surveillance. Onkologie.

[3] Li J, Xu D, Li RY (2020). Dynamic change in serum alpha-fetoprotein level predicts treatment response and prognosis of alpha-fetoprotein-producing gastric cancer. Medicine (Baltimore).

[4] Iseki M, Suzuki T, Koizumi Y (1986). Alpha-fetoprotein-producing pancreatoblastoma. A case report. Cancer.

[5] Bei R, Mizejewski GJ (2011). Alpha fetoprotein is more than a hepatocellular cancer biomarker: from spontaneous immune response in cancer patients to the development of an AFP-based cancer vaccine. Curr Mol Med.

[6] Corlin RF, Tompkins RK (1972). Serum alpha 1-fetoglobulin in a patient with hepatic metastases from bronchogenic carcinoma. Am J Dig Dis.

[7] Asamura H, Nakayama H, Kondo H (1996). AFP-producing squamous cell carcinoma of the lung in an adolescent. Jpn J Clin Oncol.

[8] Hiroshima K, Iyoda A, Toyozaki T (2002). Alpha-fetoprotein-producing lung carcinoma: report of three cases. Pathol Intern.

[9] Liu M, Liu B, Zhou YH (2016). AFP-producing lung squamous carcinoma. QJM.

[10] Chen J, Li J, Xu Y (2016). A case report of primary lung squamous cell carcinoma with abnormally increased AFP. Zhonghua Zhong Liu Fang Zhi Za Zhi.

[11] Okunaka T, Kato H, Konaka C (1992). Primary lung cancer producing alpha-fetoprotein. Ann Thorac Surg.

[12] Han B, Li K, Wang Q (2018). Effect of anlotinib as a third-line or further treatment on overall survival of patients with advanced non-small cell lung cancer: The ALTER 0303 Phase 3 Randomized Clinical Trial. JAMA Oncol.

[13] Cheng Y, Wang Q, Li K (2018). Anlotinib as third-line or further-line treatment in relapsed SCLC: A multicentre, randomized, double blind phase 2 trial. J Thorac Oncol.

[14] Basse V, Schick U, Gueguen P (2018). A mismatch repair-deficient hepatoid adenocarcinoma of the lung responding to anti-PD-L1 durvalumab therapy despite no PD-L1 expression. J Thorac Oncol.

[15] Pasricha S, Grover S, Kamboj M (2021). Hepatoid adenocarcinoma of lung: A diagnostic challenge - series of six cases with histopathological, predictive molecular and PD-L1 assessment. Indian J Pathol Microbiol.

[16] Jiang W, Huang Y, An Y (2015). Remodeling tumor vasculature to enhance delivery of intermediate-sized nanoparticles. ACS Nano.

[17] Allen E, Jabouille A, Rivera LB (2017). Combined antiangiogenic and anti-PD-L1 therapy stimulates tumor immunity through HEV formation. Sci Transl Med.

[18] Ramjiawan RR, Griffioen AW, Duda DG (2017). Anti-angiogenesis for cancer revisited: is there a role for combinations with immunotherapy?. Angiogenesis.

[19] Yang S, Zhang W, Chen Q (2020). Clinical investigation of the efficacy and safety of anlotinib with immunotherapy in advanced non-small cell lung cancer as third-line therapy: A retrospective study. Cancer Manag Res.

